# A suppressor screen of an *Arabidopsis thaliana* *REDUCED COMPLEXITY* (*RCO*)‐expressing strain provides insight into the genetics of leaf margin complexity

**DOI:** 10.1111/tpj.70278

**Published:** 2025-06-13

**Authors:** Yi Wang, Neha Bhatia, Miltos Tsiantis

**Affiliations:** ^1^ Department of Comparative Development and Genetics Max Planck Institute for Plant Breeding Research Carl von Linne Weg 10 50829 Cologne Germany; ^2^ Present address: Institute for Plant Cell Biology and Biotechnology Heinrich‐Heine‐University Düsseldorf Universitätsstr. 1, 40225 Düsseldorf Germany; ^3^ Present address: Sainsbury Laboratory Cambridge University 47 Bateman Street Cambridge CB2 1LR UK

**Keywords:** leaf margin, leaf complexity, *Cardamine hirsuta*, *REDUCED COMPLEXITY*, mutagenesis screen, *CYP71*

## Abstract

The varied leaf morphologies of seed plants provide an attractive system for studying the development and evolution of biological forms. Here, we consider the genetic mechanisms underlying variation in leaf margin geometry, as leaves can bear protrusions ranging from shallow serrations to lobes to fully separated leaflets. Leaflet formation in the complex‐leaved species *Cardamine hirsuta* requires the *REDUCED COMPLEXITY* (*RCO*) homeobox gene. *RCO* was lost in the lineage of its simple‐leaved relative *Arabidopsis thaliana*, and re‐introduction of *ChRCO* into *A. thaliana* as a transgene increases leaf complexity by triggering the generation of deep lobes in the leaf margin. As the genetic mechanisms for *RCO*‐mediated outgrowth formation are only partially understood, we performed a mutagenesis screen for suppressors of lobe formation in *A. thaliana* plants harboring a *ChRCO* transgene. From this screen, we identified *CUP‐SHAPED COTYLEDON 2* (*CUC2*), *PIN‐FORMED 1* (*PIN1*), *CYCLOPHILIN 71* (*CYP71*), *NUCLEOLAR PROTEIN 2A* (*NOP2A*), *RIBOSOMAL PROTEIN L34* (*RPL34*), and *RIBOSOMAL PROTEIN L10aB*/*PIGGYBACK1* (*PGY1*). We also showed that the *C. hirsuta CYP71* gene is required for leaflet development, as the *cyp71* mutant has simplified leaves. Our results suggest that CUC2‐auxin‐PIN1‐mediated marginal patterning, the *CYP71* gene, and ribosome biogenesis are required for *RCO* to drive increased leaf complexity.

## Significance Statement

One key regulator of leaf complexity in crucifers is the *RCO* gene, but its mechanism of action is not fully understood. A mutagenesis screen of transgenic *A. thaliana* expressing *RCO* from its complex‐leaved relative *Cardamine hirsuta* demonstrates that CUC2‐auxin‐PIN1‐mediated marginal patterning, the *CYP71* gene, and ribosome biogenesis are required for *RCO* to exert its function in leaf complexity.

## INTRODUCTION

**Figure 1:**
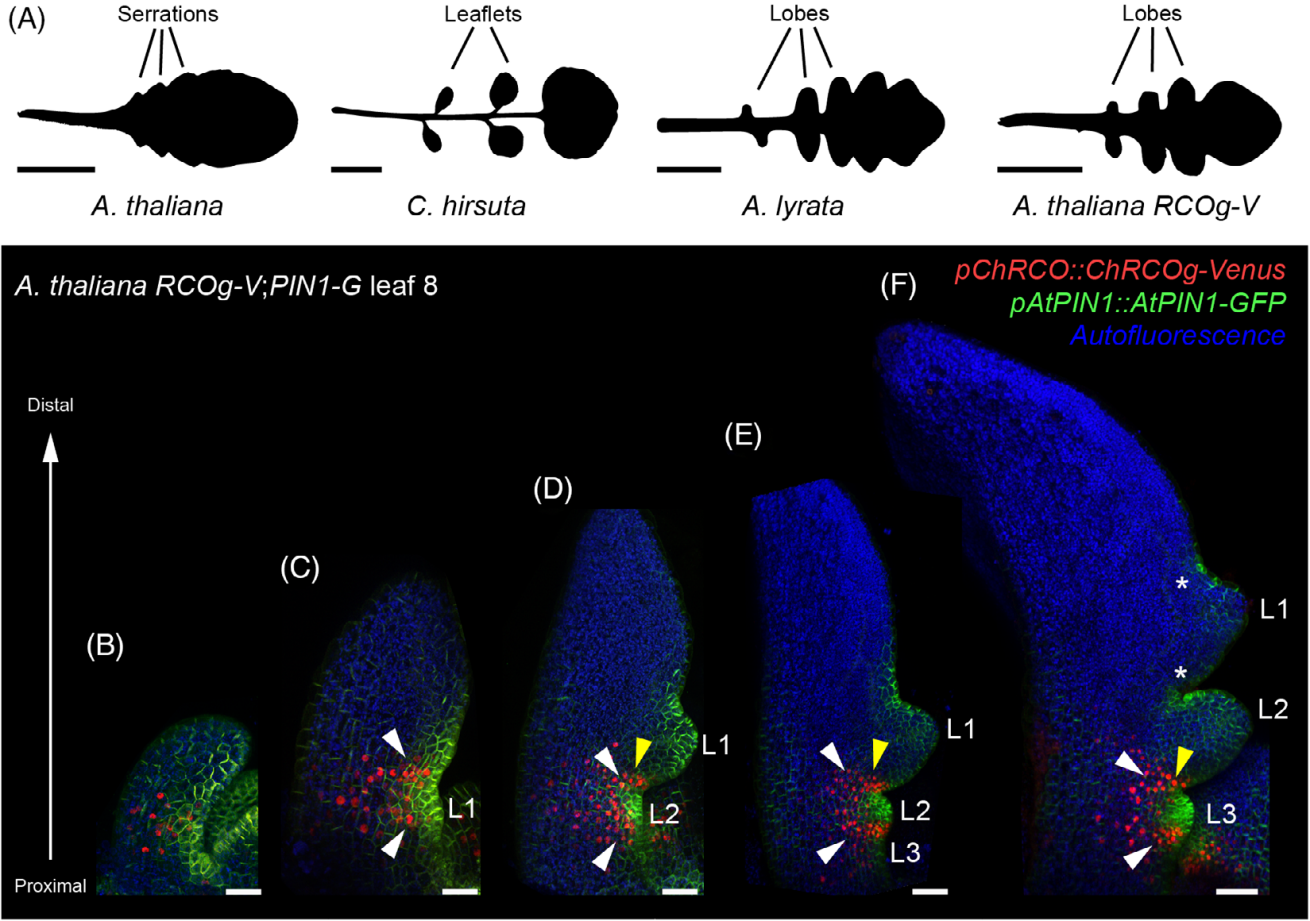
Spatio‐temporal distribution of *RCO* expression in *Arabidopsis thaliana* lobed leaf development. (A) Silhouettes of rosette leaves from *A. thaliana* wild type, *Cardamine hirsuta* wild type, *Arabidopsis lyrata* wild type (image from (Piazza et al., 2010)) and *A. thaliana pChRCO::ChRCOg‐VENUS* (*RCOg‐V*) mutant. Characteristic leaf marginal outgrowths (serrations, leaflets and lobes) are indicated. (B–F) Representative confocal projections of *A. thaliana RCOg‐V*;*pAtPIN1::PIN1‐GFP* (*PIN1‐G*) leaf 8 at progressive developmental stages. Images show RCOg‐V in red, PIN1‐G in green, and chlorophyll autofluorescence in blue. L, lobes. Note the presence of *RCOg‐V* expression (white arrowheads) flanking the initiating lobes (L1 in (C), L2 in (D, E) and L3 in (F)). As lobes start to outgrow, *RCOg‐V* expression becomes restricted to the proximal side of the lobe base (yellow arrowhead for L1 in (D, E) and L2 in (F)). *RCOg‐V* expression is not detected at the lobe base in later stages of lobe development (asterisks for L1 in (F)). Leaf nodes 6, 7, or 8 were imaged at different developmental stages with similar results (*n* = 2 per developmental stage). Scale bars: 1 cm (A), 20 μm (B–E), 30 μm (F).

Leaves of seed plants provide an attractive system to study the development and evolution of form, as they show a tremendous degree of heritable morphological variation. An example of this variation is seen in the geometry of leaf margins, which can bear protrusions of different sizes and shapes. These protrusions in the simple‐leaved *Arabidopsis thaliana* plant are shallow and are referred to as teeth or serrations (Figure 1A). Contrastingly, in fully dissected leaves like those of the *A. thaliana* relative *Cardamine hirsuta*, marginal outgrowths separate into distinct leaflets, generating a complex leaf shape (Bhatia et al., 2021; Canales et al., 2010) (Figure 1A). In other *Arabidopsis* species (e.g., *Arabidopsis lyrata*), protrusions along the leaf margin form lobes with deep sinuses (Piazza et al., 2010), a state that can be considered intermediate between leaflets and serrations (Figure 1A).

Comparison of the pathways governing simple versus complex leaf development in *A. thaliana* and *C. hirsuta* has provided considerable insights into the genetic basis and evolution of leaf shape in crucifers (Bhatia et al., 2021; Hay & Tsiantis, 2016). Notably, previous work has suggested that the gene *REDUCED COMPLEXITY* (*RCO*) had a key role in the evolution of leaf complexity. *RCO* encodes an HD‐ZIP I transcription factor, which is expressed in *C. hirsuta* leaves and is required for leaflet formation. The *RCO* gene evolved in crucifers through gene duplication, and although it was secondarily lost from the *A. thaliana* genome, re‐introducing *ChRCO* into *A. thaliana* as a transgene is sufficient to increase leaf complexity and induce deep lobes in the leaf margin (Vlad et al., 2014). In *C. hirsuta*, RCO function is partly mediated by the plant hormone cytokinin (Hajheidari et al., 2019); however, elevated cytokinin signaling in the *RCO* expression domain can only partially substitute for *RCO* in the generation of leaf complexity (Hajheidari et al., 2019). Thus, it is still not clear how RCO function is regulated at the molecular level, and which genetic mechanisms interact with RCO to regulate increased marginal complexity.

Irrespective of size or shape, the formation of protrusions at the leaf margin happens in two phases: an initial patterning phase in which protrusion primordia are initiated, followed by blade growth (a post‐patterning process), which gives protrusions their final geometry in mature leaves (Hagemann & Gleissberg, 1996; Kierzkowski et al., 2019; Koenig et al., 2009). Current evidence indicates that the patterning phase requires the plant hormone auxin, its transport protein PIN‐FORMED 1 (PIN1) and the transcription factor CUP‐SHAPED COTYLEDON 2 (CUC2) in *A. thaliana*. Specifically, auxin, CUC2, and PIN1 form interlinked feedback loops to generate interspersed foci of auxin activity maxima and CUC2 expression at the leaf margin, where outgrowths and indentations will form (Bilsborough et al., 2011). In *C. hirsuta*, ChCUC1 (a paralog of CUC2) activates the transcription of *WAG* kinases and regulates the polarity of PIN proteins during patterning (Hu et al., 2024). Although *RCO* is expressed later than *ChCUC2* during leaflet initiation and is proposed to act predominantly in post‐patterning processes to shape leaf form (Bhatia et al., 2023), whether the CUC2‐auxin‐PIN1 module is obligatory for RCO‐mediated lobe formation in leaves, and how it interacts with the RCO pathway, has not been investigated.

To identify pathways that genetically interact with RCO, in an unbiased fashion, we re‐introduced *RCO* (as *ChRCO*) into the genome of *A. thaliana* and conducted a mutant screen for suppressors that reduce the formation of lobed leaves in these plants. We found that CUC2‐auxin‐PIN1‐mediated marginal patterning, the *CYCLOPHILIN 71* (*CYP71*) gene, and ribosome biogenesis are required for *RCO* function in leaf complexity.

## RESULTS

### *RCO* acts post‐patterning and is transiently expressed during lobed leaf formation in an *A. thaliana RCO‐*expressing strain

Introduction of *ChRCO* (as *RCOg* (Vlad et al., 2014) or *pChRCO::ChRCOg‐VENUS*, “*RCOg‐V*”) into the genome of *A. thaliana* causes the development of lobed leaves rather than simple leaves with serrated margins (Hajheidari et al., 2019). To understand the spatio‐temporal distribution of *ChRCO* expression during periodic lobe emergence in the *A. thaliana RCOg‐V* leaf margin, we monitored the expression of *RCOg‐V* and *pAtPIN1::PIN1‐GFP* (*PIN1‐G*, which marks sites of protrusion emergence (Benková et al., 2003)) at progressive developmental stages of *RCOg‐V* leaf primordia (Figure 1B–F). While RCO‐VENUS was detected sporadically at leaf primordium bases prior to lobe emergence (Figure 1B), expression became discretely organized in subsequent developmental stages, flanking the emerging lobes (which are marked by a strong PIN1‐G signal with polarity convergence) (Figure 1C,D). As the lobes grew out, *RCOg‐V* expression became restricted to the proximal side of the growing lobe base and was absent from the distal side (Figure 1E,F). *RCOg‐V* expression was not detected at either side of the lobe base at later stages of lobe development (Figure 1F). Together, these observations indicate a post‐patterning action of *RCO*, whose expression is discrete along *A. thaliana RCOg‐V* leaf margins during lobe formation. This is similar to *RCO* expression during lateral leaflet formation in *C. hirsuta* (Bhatia et al., 2023); however, while *RCO* expression in *A. thaliana RCOg‐V* leaves is transient during lobe formation, *RCO* in *C. hirsuta* is expressed in leaflet bases until rosette leaves are mature (Vlad et al., 2014).

### *Suppressors of lobes* (*slb*) mutants show reduced leaf lobe phenotypes

**Figure 2:**
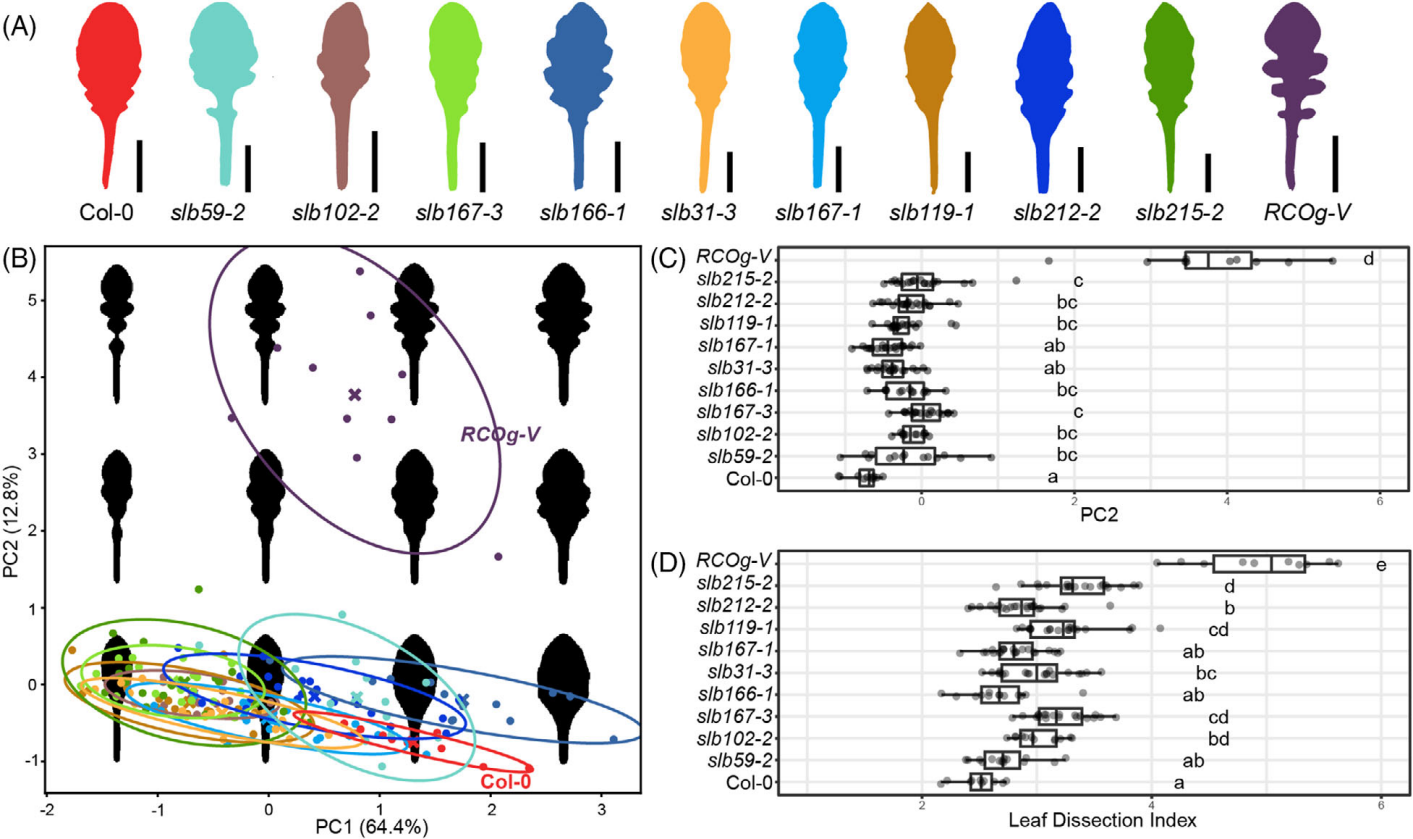
*Suppressors of lobes* (*slb*) mutants show reduced leaf lobe formation compared to *RCOg‐V*. (A) Representative silhouettes of the 10th rosette leaf from *A. thaliana* wild‐type Col‐0, *slb59‐2*, *slb102‐2*, *slb167‐3*, *slb166‐1*, *slb31‐3*, *slb167‐1*, *slb119‐1*, *slb212‐2*, *slb215‐2*, and *RCOg‐V* plants (1‐month‐old). (B) Leaf shape space analysis for the 10th rosette leaves from the genotypes shown in (A) using Leaf Interrogator (Zhang et al., 2020). Genotypes are colored as in (A). The crosses and eclipses indicate the mean value and standard deviation of each genotype, respectively. The plot shows the first two Principal Components (PCs) of the observed variance. The value of PC2 (12.8% of the total variance) captures the formation of lobes in leaf shapes, whereas the value of PC1 (64.4% of the total variance) captures the ratio of leaf length to width. The black silhouettes are reconstructed from the principal components to visualize the variation captured by the shape space. (C) Box plots to compare the PC2 values of corresponding genotypes in the leaf shape space of (B). (D) Box plots to compare the leaf dissection indexes of corresponding genotypes. Compact letters in panels (C) and (D) indicate significant differences (ANOVA combined with Tukey HSD tests, *P* < 0.05). *n* = 10–20 per genotype. Scale bars: 1 cm (A).

Having characterized the expression of *RCO* in the context of auxin‐based patterning of margin protrusions in *A. thaliana RCOg‐V*, we next sought to identify genes required for *RCO*‐mediated leaf complexity in this species. To this end, we screened ethyl methanesulfonate (EMS) mutagenized homozygous *A. thaliana RCOg‐V* plants (Wang et al., 2022), aiming for suppressors, that is, mutants showing simplified leaves and reduced lobe formation. We identified nine suppressors and named these *suppressors of lobes* (*slb* mutants) (Figure 2A). To quantify and compare the leaf shape geometries of these *slb* mutants to *A. thaliana RCOg‐V* and wild‐type plants, we conducted both univariate (leaf dissection index) and multivariate shape analyses (Leaf Interrogator) (Zhang et al., 2020). These analyses confirmed a significant reduction in lobe formation and leaf shape complexity in the *slb* mutants compared to *RCOg‐V* (Figure 2B–D).

### Mapping‐by‐sequencing and allelism analysis of the *slb* mutants

**Figure 3:**
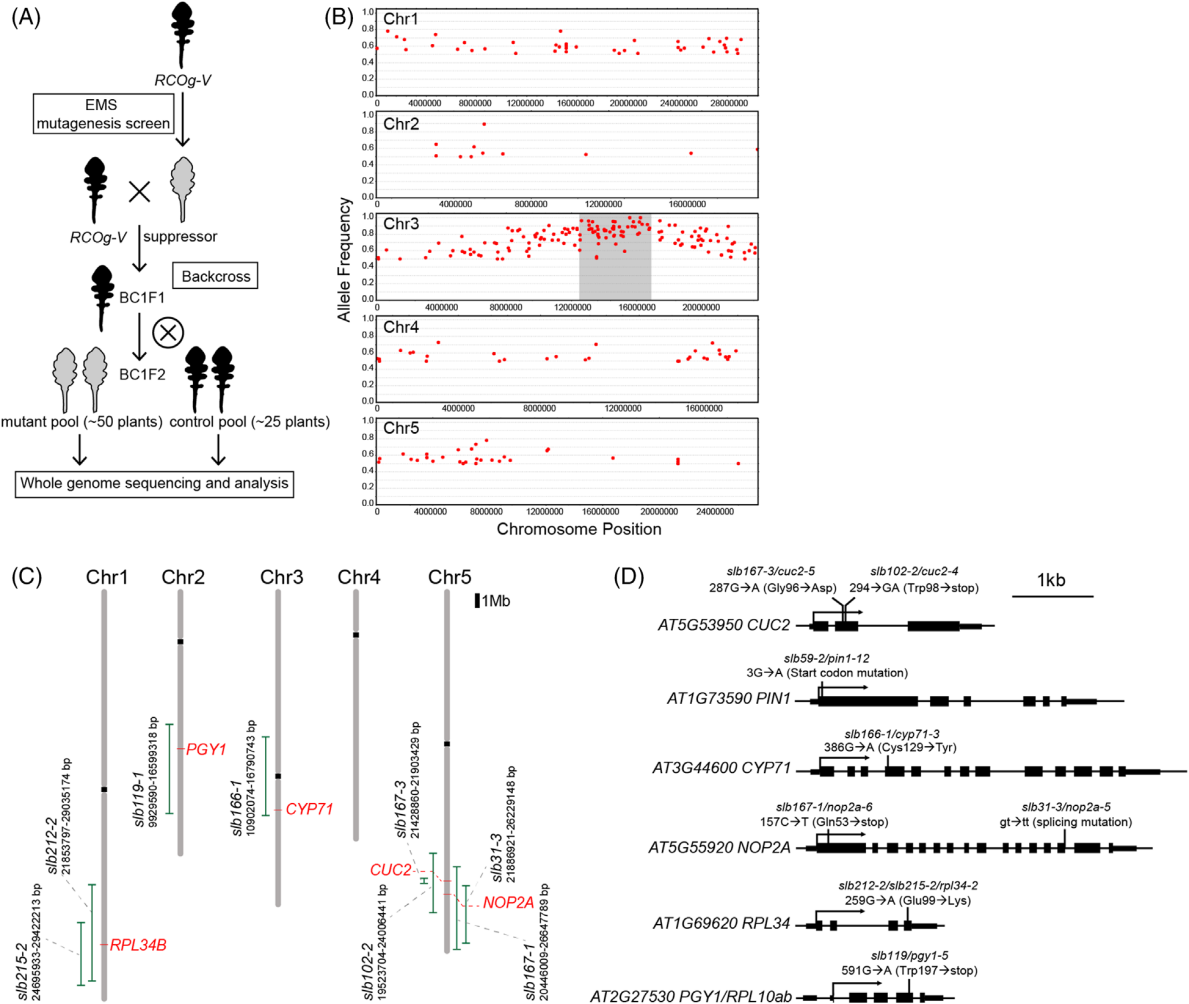
Mapping‐by‐sequencing to identify causal mutations in *slb* mutants. (A) Workflow of mapping‐by‐sequencing. Backcrossed F2 populations of each suppressor were used for the experiment. (B) An example of allele frequency plots from the *slb166‐1* mutant pool. Red points show polymorphisms from EMS‐induced mutations. A trend of high allele frequency can be observed in the center of chromosome 3. The causal *locus* was mapped to an interval (the gray region) in chromosome (Chr) 3 where both ends contain polymorphisms in >90% of reads. (C) Distribution of mapping intervals (green lines) of *slb* mutants on *A. thaliana* Chromosomes 1–5. Candidate responsible genes are labeled in red color. (D) Schematic representations of gene structures to show the mutations and corresponding amino acid/mRNA structure changes (in brackets) of the *slb* mutants. Exons are drawn as black boxes and UTRs as flat boxes.

To identify the causal mutations for the *slb* mutants, we performed mapping‐by‐sequencing (Leshchiner et al., 2012; Schneeberger, 2014). First, to understand whether the *slb* phenotypes were caused by single or multiple loci of dominant or recessive mutations, we backcrossed (BC) the *slb* mutants to *RCOg‐V* plants and phenotyped the F1 and F2 populations. All BC1F1 plants showed a lobed‐leaf phenotype, and BC1F2s displayed a 3:1 segregation ratio of plants with lobed or simple leaves. This suggests that each suppressor mutant likely represents a single recessive locus. To map the chromosomal locations of these mutations, we subjected the BC1F2 populations (Figure 3A) to whole genome re‐sequencing and allele frequency analysis. In this way, *slb* mutations were mapped to 0.5‐7 Mb intervals in the *A. thaliana* genome (Figure 3B,C).

Based on these mapping intervals, we worked to identify candidate genes for *slb* mutations by considering linkage to genes previously known to affect leaf development, and/or the presence of single nucleotide polymorphisms (SNPs) in genes in these genetic intervals. We found that *slb102‐2* and *slb167‐3* share an overlapping interval on Chromosome 5 (Figure 3C), and within this region, both mutants carry SNPs in the *CUC2* gene that promotes serration development (Nikovics et al., 2006): a nonsense mutation in *slb102‐2* (G294 → A; Trp98 → stop); a missense mutation in *slb167‐3* (G287 → A; Gly96 → Asp) (Figure 3D). As an allelism test confirmed that *slb102‐2* and *slb167‐3* are mutants of the same gene (Table 1, Figure S1e), we predicted that their leaf phenotypes may result from mutations in *CUC2*. Another mutant, *slb59‐2*, exhibited pin‐like inflorescences, a characteristic phenotype of *pin1* loss‐of‐function mutants in which serration development is also compromised (Bilsborough et al., 2011). Correspondingly, we found that *slb59‐2* harbors a mutated start codon in *PIN1* (G3 → A; Met1 lost); therefore, we speculated that this mutation causes the *slb59‐2* leaf phenotype (Figure 3D).

**Table 1:** Allelism test results of the *slb* mutants

Parent plants		F1 phenotypes	
P1	P2	Lobed leaf	Unlobed (simplified) leaf
*slb167‐3*	*cuc2‐3* (*SAIL_605_C09*)*;RCOg‐V*	0	57
*slb102‐2*	*cuc2‐3* (*SAIL_605_C09*)*;RCOg‐V*	0	38
*slb167‐3*	*slb102‐2*	0	20
*slb59‐2*	*pin1‐7*(*+/−*)^†^ (*SALK_047613*)	20	20
*slb166‐1*	*cyp71‐2*(*+/−*)^‡^ (*SALK_050092*)	36	36
*slb31‐3*	*nop2a‐2* (*SALK_129648C*)	0	5
*slb167‐1*	*nop2a‐2* (*SALK_129648C*)	0	27
*slb31‐3*	*slb167‐1*	0	27
*slb212‐2*	*rpl34‐1* (*SALK_025693C*)	0	24
*slb215‐2*	*rpl34‐1* (*SALK_025693C*)	0	34
*slb212‐2*	*slb215‐2*	0	31
*slb119‐1*	*pgy1‐4*/*rpl10ab‐4* (*SALK_087642C*)	0	25

Each *slb* mutant bears an EMS mutation and is homozygous for the *RCOg‐V* transgene in its genome. In this allelism test, *slb* mutants were crossed with corresponding T‐DNA insertion mutants that lack the *RCOg‐V* transgene, except for *slb167‐3* and *slb102‐2*, which were crossed with a *cuc2‐3;RCOg‐V* double mutant. Some *slb* mutants were also crossed with each other to test whether they were mutant alleles of the same gene.

^†^ Heterozygous *pin1‐7 (+/−)* was used for crossing because the homozygous *pin1‐7* mutant is infertile. *slb59‐2* is fertile. F1 plants were genotyped, and only plants with the T‐DNA insertion showed an unlobed phenotype.

^‡^ Heterozygous *cyp71‐2 (+/−)* was used for crossing because the homozygous *cyp71‐2* mutant shows abnormal flowers and low fertility. *slb166‐1* is fertile. F1 plants were genotyped and only plants with the T‐DNA insertion showed an unlobed phenotype.

Next, we considered the *slb31‐3*, *slb167‐1*, *slb212‐2*, *slb215‐2*, and *slb119‐1* mutants. We found these to be associated with intervals containing genes relating to ribosomal function, which is known to influence leaf development in both *A. thaliana* and *C. hirsuta* (Byrne, 2009; Kougioumoutzi et al., 2013). The specific candidate genes and SNPs that we hypothesized may underlie these ribosomal‐related *slb* phenotypes are as follows: *NUCLEOLAR PROTEIN 2A* (*NOP2A*)/*OLIGOCELLULA2* (*OIL2*), a gene involved in ribosome biogenesis and leaf development (Fujikura et al., 2009; Kojima et al., 2018), has a splice‐site mutation (gt → tt, 14th exon) in *slb31‐3* and a nonsense mutation (C157 → T; Gln53 → stop) in *slb167‐1* (Figure 3D); we also confirmed that these two mutants are allelic (Table 1). *slb212‐2* and *slb215‐2* share the same missense mutation in *60S ribosomal protein L34* (*RPL34*) (G259 → A; Glu99 → Lys) (Figure 3D); and *slb119‐1* has a nonsense mutation (G591 → A; Trp197 → stop) (Figure 3D) in *ribosomal protein L10aB/PIGGYBACK1* (*PGY1*)—a gene known to affect *A. thaliana* leaf tissue polarity (Pinon et al., 2008). Finally, we speculated that *slb166‐1* results from a missense mutation in the *CYP71* gene (G386 → A; Cys129 → Tyr) (Figure 3D). *CYP71* encodes the WD40 domain‐containing cyclophilin 71 that is associated with chromatin and reported to influence leaf blade and vasculature development in *A. thaliana* (Lakhanpal et al., 2020; Li et al., 2007).

To test our predictions that mutations in the above‐stated genes underlie *slb* phenotypes, we conducted allelism analyses by crossing each *slb* mutant to a T‐DNA insertion mutant in which the function of the respective candidate gene is disrupted (Table 1). All F1 plants containing T‐DNA inserted alleles displayed simple rosette leaves without lobes (Table 1 and Figure S1), supporting our predictions of *slb* causal mutations. In summary, through mapping‐by‐sequencing and allelism analyses, we have identified the *CUC2*, *PIN1*, *CYP71*, *NOP2A*, *RPL34*, and *PGY1* genes as being required for lobed leaf formation in *A. thaliana RCOg‐V* plants. We subsequently renamed the *slb* mutants as *cuc2‐4*;*RCOg‐V* (*slb102‐2*), *cuc2‐5*;*RCOg‐V* (*slb167‐3*), *pin1‐12*;*RCOg‐V* (*slb59‐2*), *cyp71‐3*;*RCOg‐V* (*slb166‐1*), *nop2a‐5*;*RCOg‐V* (*slb31‐3*), *nop2a‐6*;*RCOg‐V* (*slb167‐1*), *rpl34‐2*;*RCOg‐V* (*slb212‐2* and *slb215‐2*), and *pgy1‐5*;*RCOg‐V* (*slb119‐1*) (Figure 3D).

### Lobe initiation and *RCO* expression in *slb* leaf primordia

**Figure 4:**
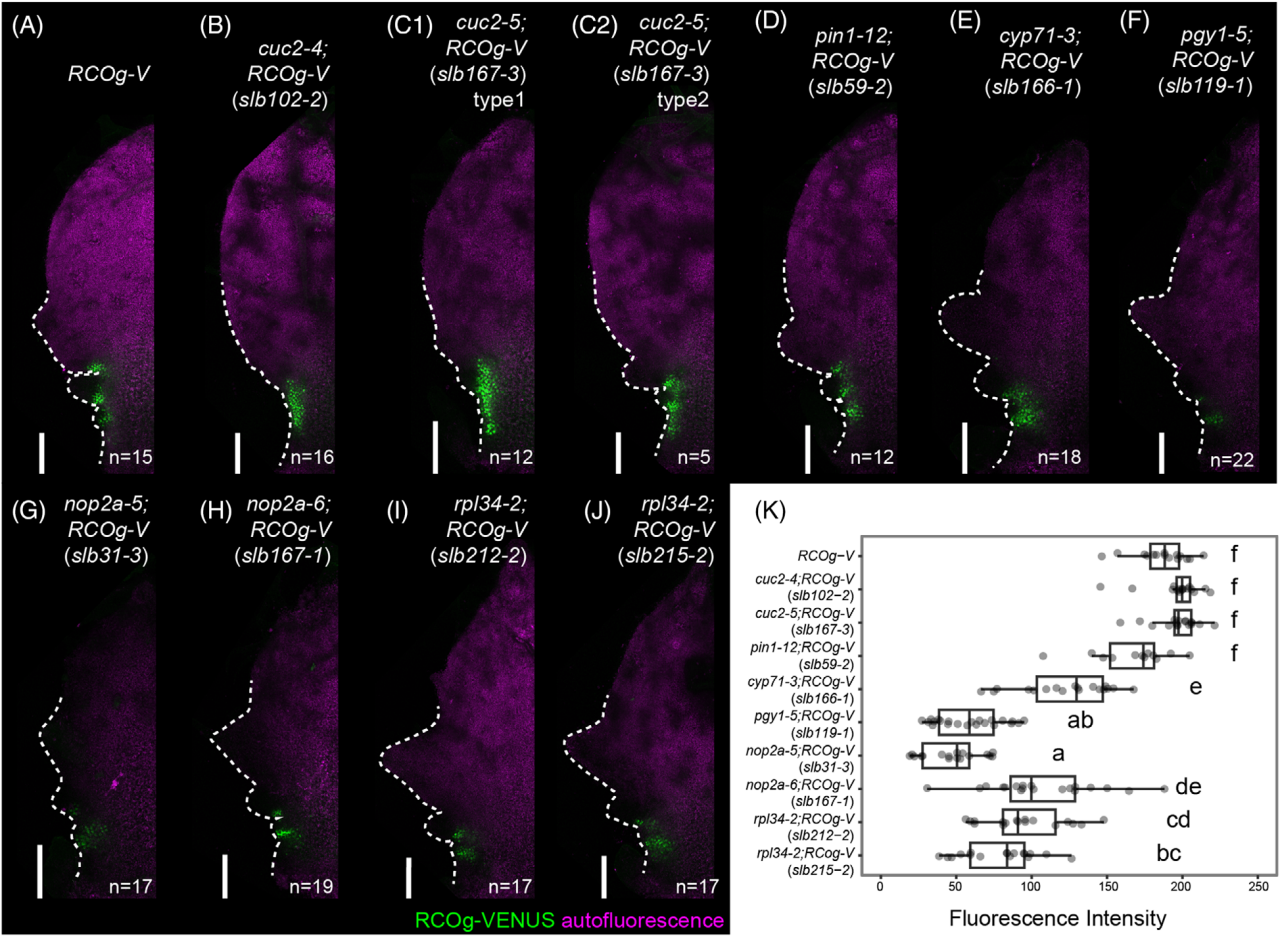
Marginal shapes and *RCO* expression patterns in *slb* leaf primordia. (A–J) Representative confocal microscopy images (maximum intensity projections) of the 7th or 8th rosette leaf primordia (600–1200 μm length) from 2‐week‐old *RCOg‐V* (A) and the *slb* mutants (B–J). RCO‐VENUS signal (green) and chlorophyll autofluorescence (magenta) are presented. (C1) and (C2) show two types of phenotypes observed in *cuc2‐5;RCOg‐V* (*slb167‐3*) samples. Dashed lines show leaf margins. (K) Box plots to compare RCO‐VENUS fluorescence intensity in the leaf primordia of *RCOg‐V* and the *slb* mutants. Fluorescence intensities were measured from the confocal microscope images represented in (A–J). Compact letters indicate significant differences (ANOVA combined with Tukey HSD tests, *P* < 0.05). Replicate numbers for each genotype are represented per image panel. Scale bars, 100 μm.

To understand the processes causing the divergent leaf marginal protrusions of the *slb* mutants, we imaged *A. thaliana RCOg‐V* and *slb* leaf primordia (600–1200 μm length) using confocal microscopy, and compared their leaf marginal shapes (Figure 4). While the leaf margins of *RCOg‐V* recurringly generate lobe primordia, *cuc2‐4;RCOg‐V* and *cuc2‐5;RCOg‐V* of a similar age display smooth leaf margins without lobe primordia (Figure 4A–C1), suggesting that lobe initiation is defective in the *cuc2;RCOg‐V* mutants. We also noted that a subset of *cuc2‐5;RCOg‐V* leaf samples (5/17) produced lobe primordia (Figure 4C2), suggesting that *cuc2‐5* (a missense mutation, Gly96 → Asp) may be a weaker allele than *cuc2‐4* (a nonsense mutation, Trp98 → stop) (Figure 3D). As all other *slb* mutants showed a pattern of lobe primordia initiation similar to *RCOg‐V* (Figure 4D–J), defects in subsequent lobe growth (post patterning) likely lead to reduced lobe phenotypes in these mutants. Taken together, our results show that *CUC2* is required for lobe initiation, while disruption of *PIN1*, *CYP71*, *NOP2A*, *RPL34*, and *PGY1* function in the alleles isolated here perturbs lobe development after lobes initiate.

We next considered whether altered lobe development in *slb* mutants is caused by regulation of *RCO* expression in leaf margins. To test this idea, we compared *RCOg‐V* expression patterns and levels in *slb* and *A. thaliana RCOg‐V* leaf primordia. In *RCOg‐V* plants, *RCOg‐V* is expressed in sinuses and lobe primordium bases along leaf margins in a non‐continuous manner (Figures 1 and 4A). This typical localized expression pattern underlies repression of local growth at the sinuses and promotes lobe formation (Bhatia et al., 2023; Vlad et al., 2014; Wang et al., 2022). Most *slb* mutants retain this pattern of non‐continuous *RCOg‐V* expression in leaf primordia, except for *cuc2‐4;RCOg‐V* and *cuc2‐5;RCOg‐V*, in which the RCO‐VENUS fluorescent signal is continuous along their smooth leaf margins (Figure 4B–J). This result is consistent with our observation that *CUC2* is required for lobe initiation and further indicates that CUC2 is necessary for patterning non‐continuous *RCO* expression along the leaf primordium margin. From this, we infer that the continuous domain of *RCO* expression in *cuc2;RCOg‐V* might prevent proper lobe initiation and growth. Notably, however, despite both CUC2 and PIN1 acting in marginal patterning (Bilsborough et al., 2011; Hu et al., 2024; Kierzkowski et al., 2019), *pin1‐12;RCOg‐V* did not display lobe initiation defects and modified *RCO* expression similar to those observed in *cuc2;RCOg‐V* as one might have predicted. One possible explanation is that *pin1‐12* is a weak allele, as *pin1‐12;RCOg‐V* plants produce siliques with seeds before pin‐like inflorescence formation (Figure S2). Another possibility is that auxin function is perturbed to different degrees in the *pin* and *cuc* mutants and that such differences influence the leaf margin phenotypes and sensitivity of *RCO* expression.

In addition, we found RCO‐VENUS fluorescence intensity to be reduced in *cyp71‐3*, *nop2a‐5*, *nop2a‐6*, *rpl34‐2*, and *pgy1‐5* compared to *RCOg‐V* (Figure 4F–K), suggesting that these genes may be required for maintaining *RCO* expression levels. Accordingly, reduced RCO levels might contribute to reduced lobe formation in leaves, though it is also possible that this reduction is an indirect effect of changes in the growth and proliferation of leaf margin tissue in these mutants.

### *C. hirsuta cyp71* mutants show simplified leaves

After determining that *CUC2*, *PIN1*, *NOP2A*, *RPL34*, *PGY1*, and *CYP71* are required for *RCO*‐mediated lobe formation in *A. thaliana* leaves, we next considered whether their orthologous genes might contribute to leaflet formation in *C. hirsuta*, the native system for *RCO* expression. Indeed, *C. hirsuta pin1* (*chpin1*) mutants are known to produce simplified leaves without leaflets (Barkoulas et al., 2008), and *C. hirsuta* mutants with reduced expression of *CUC2* and its redundant paralogues *CUC1* and *CUC3* also display simplified leaves with reduced leaflets (Blein et al., 2008). *RPL34*, *PGY1*, and *NOP2A* are related to ribosome biogenesis and function, and we have previously shown that *SIMPLE LEAF3* (*SIL3*), which encodes a ribosome‐associated protein, is required for dissected leaf development in *C. hirsuta* (Kougioumoutzi et al., 2013). Taken together, these findings suggest that *PIN1*, *CUC*, and ribosome‐related genes regulate leaf complexity in *C. hirsuta*, consistent with their abilities to suppress lobed leaf formation in *A. thaliana RCOg‐V* plants.

**Figure 5:**
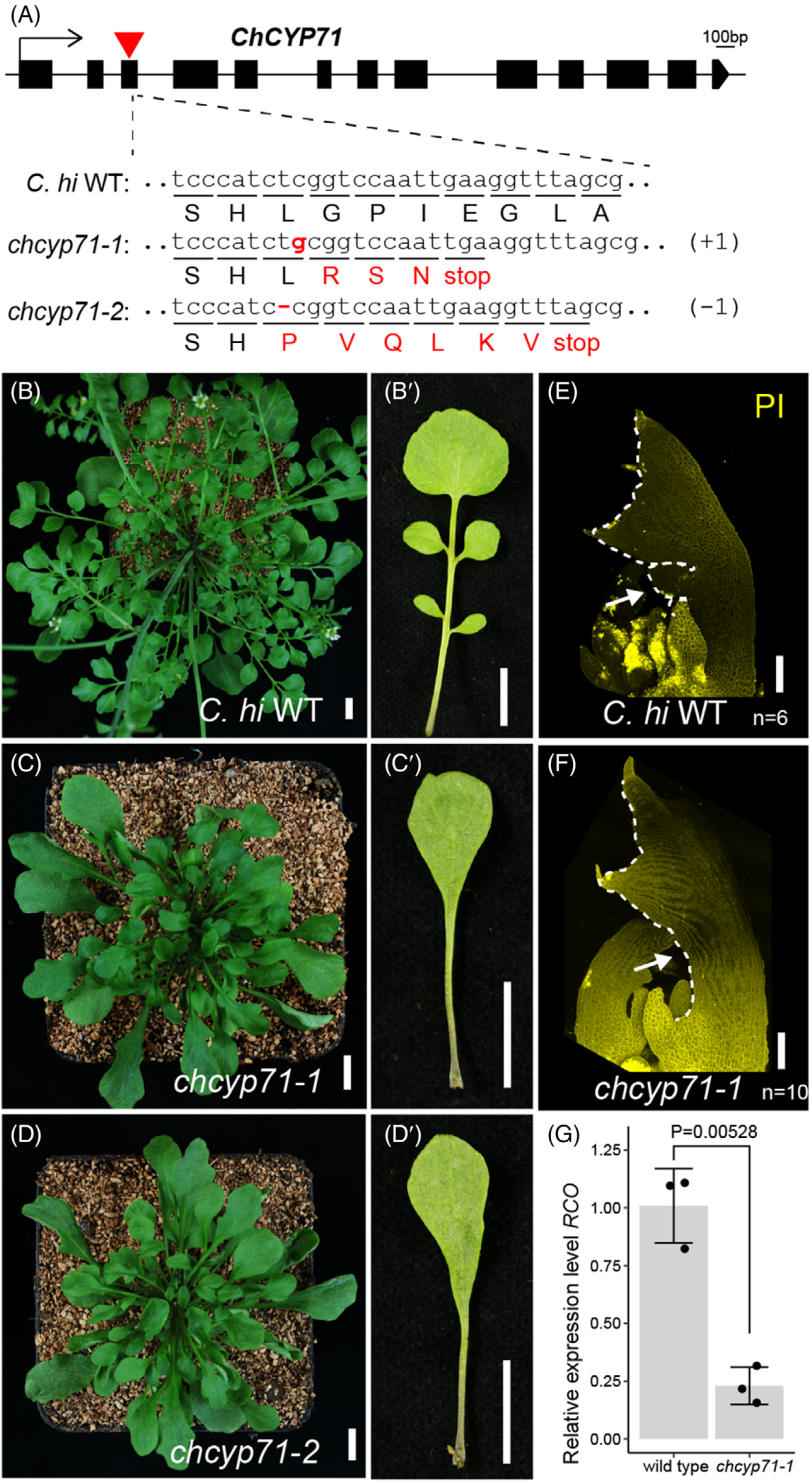
*ChCYP71* is required for dissected leaf formation in *Cardamine hirsuta*. (A) Gene structure schematics and mutations of *ChCYP71*. Exons are drawn as black boxes. The gRNA targeting site for CRISPR‐Cas9 editing is marked with red triangles. cDNA and corresponding amino acid sequences of wild type and two mutants are displayed. *chcyp71‐1* has one nucleotide insertion and *chcyp71‐2* has one nucleotide deletion. Both mutations cause a frameshift and early translation termination (labeled in red color). (B–D′) 6‐week‐old rosettes (B–D) and the 5th rosette leaves (B′–D′) from *C. hirsuta* wild type, *chcyp71‐1*, and *chcyp71‐2* plants. *n* > 20 observed per genotype. (E) and (F) Leaf primordia of *C. hirsuta* wild type (E) and *chcyp71‐1* (F). The 5th and 6th rosette leaf primordia (~ 800 μm length) from 3‐week‐old plants were imaged. Leaf cells were visualized by propidium iodide (PI, yellow). Dashed lines show leaf margins. Arrows indicate a lack of lateral leaflet primordia in *chcyp71‐1* compared to wild type. (G) Quantitative real‐time PCR showing a reduced *RCO* transcript level in *chcyp71‐1* compared to wild type (*P* = 0.00528, Welch two‐sample *t*‐test). Bar charts and error bars represent mean values and standard deviations, respectively. *n* = 3 biological replicates for each genotype. Scale bars: 1 cm (B–D'); 100 μm (E, F).

In contrast, *CYP71* has not previously been reported to regulate leaf complexity. Thus, to test whether *CYP71* has a role in complex leaf formation, we generated *cyp71* mutants in *C. hirsuta* (*chcyp71*) using the CRISPR/Cas9 method (Alvim Kamei et al., 2020). We obtained two *chcyp71* alleles: *chcyp71‐1* with one nucleotide insertion and *chcyp71‐2* with one nucleotide deletion in the coding region. Both mutations cause frameshifts and early translation termination (Figure 5A). Compared to wild type, *chcyp71‐1* and *chcyp71‐2* showed no leaflets in mature rosette leaves (Figure 5B–D’). Leaf primordia (~800 μm length) in *chcyp71‐1* also displayed a lack of lateral leaflet formation (Figure 5E,F). As these results suggest that *CYP71* is indeed required for dissected leaf formation in *C. hirsuta*, we then asked whether CYP71 also influences *RCO* expression in *C. hirsuta*, as in *A. thaliana* (Figure 4E). We compared *RCO* transcript levels in *chcyp71‐1* and wild‐type leaf primordia using quantitative real‐time PCR and found that *chcyp71‐1* samples showed reduced levels of *RCO* transcripts (Figure 5G). From this, we conclude that ChCYP71 can influence *RCO* transcription during dissected leaf formation.

## DISCUSSION

Via an EMS‐based modifier screen of *A. thaliana RCOg‐V* transgenic plants, which produce lobed leaves, we have identified mutants of *CUC2*, *PIN1*, *CYP71*, *NOP2A*, *RPL34*, and *PGY1* as suppressors of lobe formation (Figures 2 and 3). Using the same *RCOg‐V* transgenic plants, we have previously reported an enhancer screen for more complex leaf phenotypes (i.e., leaves bearing leaflets), which identified *asymmetric leaves 1* (*as1*) and *as2* mutants that regulate Class I *KNOTTED1‐LIKE HOMEOBOX* (*KNOX1*) expression (Wang et al., 2022). Comparing these studies, it is notable that more genes, with more varied biological functions, were identified from the suppressor screen. Thus, it appears easier for *A. thaliana RCOg‐V* leaves to lose lobes than to develop lobes into leaflets, suggesting that lobe development is vulnerable to diverse genetic perturbations. This is consistent with a previous report showing that flowering plants tend to retain and revert to simple leaves rather than complex leaves during evolution (Geeta et al., 2012). Our data provide a possible explanation for this trend by suggesting that the formation of complex leaves requires the action of multiple genetic pathways that influence growth and development at different levels. Compromising the function of any of these pathways may suppress lobe formation. The role of *PIN1* and *CUC2* in the development of serrations and leaflets has already been studied in some detail (Barkoulas et al., 2008; Bilsborough et al., 2011; Hu et al., 2024; Israeli et al., 2019; Koenig et al., 2009) and in the future, it will be important to also investigate the function of *CYP71*, *NOP2A*, *RPL34*, and *PGY1* in *A. thaliana* serration development, as this will enhance our comparative understanding of the development of serrations, leaflets, and lobes.

The domain of *RCO* expression during the lobed leaf formation in *A. thaliana RCOg‐V* plants is similar but transient compared to the sustained expression of *RCO* in *C. hirsuta* leaves required to drive leaflet formation (Figure 1) (Bhatia et al., 2023; Vlad et al., 2014). This difference in the duration of *RCO* expression might contribute to the formation of lobed leaves in *A. thaliana RCOg‐V* plants versus dissected leaves in *C. hirsuta*. Specifically, the absence of *RCO* expression from the bases of developing protrusions may limit the growth repression that is required for leaflet formation (Kierzkowski et al., 2019). This hypothesis is consistent with our previous findings that the expansion of *RCOg‐V* expression to the distal side of growing protrusion bases in *A. thaliana* leaves containing ectopic *KNOX1* expression causes the formation of leaflet‐like structures instead of lobes (Wang et al., 2022). Future work will be required to understand the processes underlying persistent *RCO* expression in *C. hirsuta* emerging leaflets compared to those governing transient *RCOg‐V* expression in *A. thaliana RCOg‐V* emerging lobes. Such work will also contribute to understanding similarities vs. differences in the morphogenesis of *A. thaliana RCOg‐V* lobes and *C. hirsuta* leaflets.

**Figure 6:**
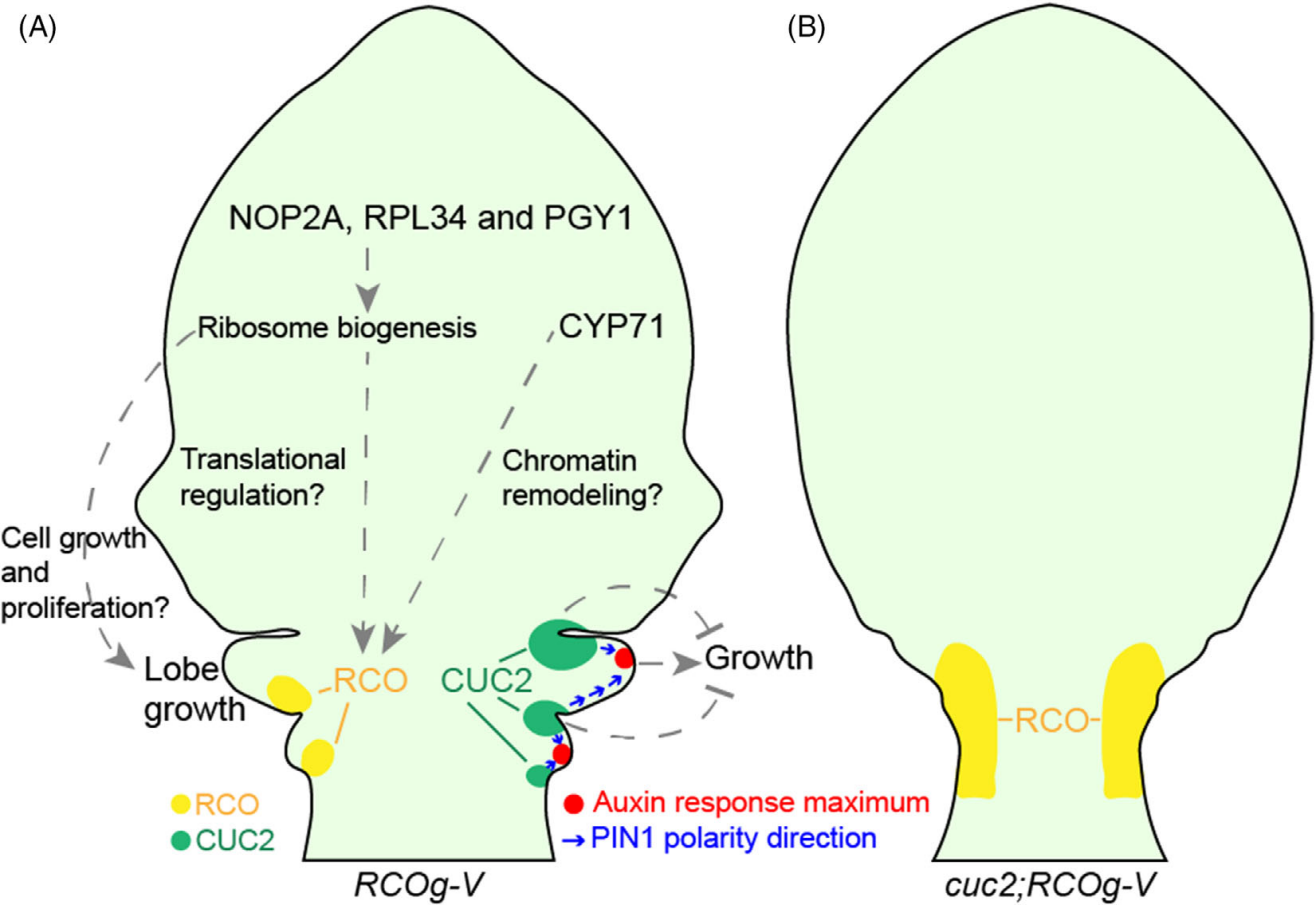
Leaf lobe formation requires the functions of PIN1, CUC2, CYP71, NOP2A, RPL34, and PGY1. Schematic showing the roles of PIN1, CUC2, CYP71, NOP2A, RPL34, and PGY1 in RCO‐mediated leaf lobe formation. In *RCOg‐V* leaf primordia (A), the CUC2‐auxin‐PIN1 module is necessary for lobe initiation and subsequent blade growth, where auxin activity promotes lobe outgrowth and CUC2 represses sinus growth. CUC2 is also required for non‐continuous *RCO* expression in lobe bases along leaf margins. Consequently, in the *cuc2;RCOg‐V* leaf primordium (B), the *RCO* expression pattern is changed, becoming continuous in the basal leaf margin. Additional regulation of RCO activity may occur via CYP71 influencing *RCO* transcription through chromatin remodeling (A), and NOP2A, RPL34, and PGY1, which are related to ribosome biogenesis, may affect RCO protein translation. These ribosome‐related genes may also influence cell growth and proliferation more broadly, and thus affect lobe growth and leaf margin shape independently of RCO (A).

Previous studies have suggested that the CUC2‐auxin‐PIN1 module is required for leaf marginal outgrowth patterning (Bilsborough et al., 2011; Hu et al., 2024; Kierzkowski et al., 2019). As we obtained *cuc2* and *pin1* mutants in our suppressor screen, our data suggest that the CUC2‐auxin‐PIN1 module is necessary for *RCO*‐mediated lobe formation, likely by regulating both lobe initiation (the patterning process) and subsequent lobe growth at the leaf margin (Figures 4B–D and 6A) (Hu et al., 2024). We also observed an association of reduced lobe initiation with continuous *RCO* expression in *A. thaliana cuc2* mutants (Figures 4B, C1 and 6B). This is consistent with recent work showing how near‐continuous *RCO* expression along the *C. hirsuta* leaf margin (resulting from precociously expressing *RCO* in the *CUC2* domain) abolishes marginal patterning and lateral leaflet formation (Bhatia et al., 2023), and highlights the significance of typical localized *RCO* expression for complex leaf development.

Chromatin remodeling can affect the action of many genes in plant development, including those involved in meristem establishment, lateral organ development, flowering, and seed germination (Ojolo et al., 2018). In *A. thaliana*, CYP71 has been reported to influence chromatin remodeling by interacting with histone H3 and suppressing the transcription of genes including the leaf shape regulator *KNOX1* (Lakhanpal et al., 2020; Li et al., 2007; Li & Luan, 2011). Our finding that *CYP71* is required for dissected leaf formation and *RCO* expression in leaf primordia (Figures 4K, 5G and 6A) raises the possibility that *CYP71* regulates *RCO* levels to shape leaf margin complexity. *ChCYP71* is also likely to regulate leaf shape development in *C. hirsuta* independent of *RCO*, as *chcyp71* mutant rosette leaves are simpler than the lobed leaves of a *chrco* mutant (Vlad et al., 2014) (Figure 5C,D’).

Ribosome‐related genes have been reported to regulate multiple aspects of leaf shape development. For example, *A. thaliana nop2a* and other mutants defective in ribosome‐related genes can enhance the leaf polarity defects of *as1* and *as2*, turning flat blades into needle‐like structures (Horiguchi et al., 2011; Kojima et al., 2018; Yao et al., 2008). Additionally, *pgy1* can promote ectopic lamina outgrowths in an *as1* background (Pinon et al., 2008). Our results show that mutations in *RPL34*, *PGY1*, and *NOP2A* genes, which are related to ribosome biogenesis, can suppress lobe formation in *A. thaliana RCOg‐V*. Although we have not tested the function of *RPL34*, *PGY1*, and *NOP2A* genes in *C. hirsuta*, *C. hirsuta SIL3* is required for leaflet development (Kougioumoutzi et al., 2013), indicating that ribosome‐related genes have a role in leaf complexity. One possible explanation is that reduced ribosome biogenesis in these mutants delays the translation of *RCO* and other necessary genes for lobe/leaflet development, leading to leaf shape simplification (Figures 4K and 6A). It is also possible that these ribosome‐related genes broadly influence cell growth and proliferation in leaves (Fujikura et al., 2009), and thus their effect on the growth of leaf margin protrusions and complex leaf forms is independent of *RCO* (Kierzkowski et al., 2019) (Figure 6A). In the future, it will be interesting to test these hypotheses and also study the cellular basis for the function of *RPL34*, *PGY1*, and *NOP2A* in both *A. thaliana* and *C. hirsuta* leaf development.

Our work also highlights the power of comparative genetics, in particular, the use of interspecies gene transfer as a method to understand the genetic basis for evolutionary changes (Nikolov & Tsiantis, 2015). Using *A. thaliana RCOg‐V* transgenic lines simplified our genetic studies of *RCO* (including mutagenesis screen and mapping) as *A. thaliana* has a smaller genome size and shorter life cycle than *C. hirsuta* (Hay et al., 2014). It further allowed an investigation of how processes that are potentially conserved between *A. thaliana* and *C. hirsuta* may affect *RCO* function in complex leaf development. This approach helped us to identify the CUC2‐auxin‐PIN1 module as an obligatory prerequisite for *RCO* action, highlighted *CYP71* as a novel component of leaflet developmental processes, and underlined the role of ribosome‐related genes in complex leaf development. In the future, it will be interesting to understand how localized *RCO* expression is recruited to the leaf margin after patterned outgrowth regulated by the CUC2‐auxin‐PIN1 module, and the precise mechanisms through which *CYP71* and ribosome‐related genes influence leaf development.

## EXPERIMENTAL PROCEDURES

### Plant material and growth conditions

Soil‐grown plants were cultivated in greenhouses under long‐day conditions (16‐h light: 8‐h dark, with supplemental lighting when natural light intensity was below 75 μmol m^−2^ s^−1^) at 22°C, or cultivated in climate chambers (Reftech) under long‐day conditions [16‐h light (20°C):8‐h dark (18°C), light intensity 110 mmol m‐2 s‐1, humidity 65%]. *A. thaliana* seeds were cold‐stratified within 1/1000 agar solutions (w/v) at 4°C for 2 days, then pipetted to a wet soil surface for germination. *C. hirsuta* seeds were sown on a wet soil surface, cold‐stratified at 4°C for 1 week, and then grown in the greenhouse. For imaging *A. thaliana* transgenic lines carrying *pChRCO::ChRCOg‐VENUS* and *pAtPIN1::AtPIN1‐GFP*, seeds were surface sterilized in 70% ethanol for 15 min, sown on square petri dishes with GM medium (per liter‐ 10 g sucrose (Sigma‐Aldrich, Cat# 84097), 4.33 g Murashige and Skoog basal salt mixture (Sigma‐Aldrich, Cat# M5524), 0.5 g MES (Roth, Cat# 4256.4), 8 g Bacto Agar (Roth Cat# 5210.5), 1 mL MS vitamins (Sigma‐Aldrich, Cat# M3900), pH 5.7 with 1 M potassium hydroxide solution) and stratified at 4°C for 2 days. Plates were transferred to growth chambers under long‐day conditions (16‐h light: 8‐h dark, light intensity ∼80 μmol m‐2 s‐1) at 18°C.

### EMS mutagenesis screen and mapping

Similar to (Vlad et al., 2014), *RCOg‐V* transgenic plants carry a *pChRCO::ChRCOg‐VENUS* cassette of the 3229 bp *RCO* promoter, the 1689 bp *RCO* genomic DNA (no stop codon) fused to C‐terminal *VENUS*, and an *OCS* terminator transformed into *A. thaliana* Col‐0 ecotype. EMS mutagenesis was performed in homozygous *RCOg‐V* transgenic seeds by treating the seeds with 100 mM ethyl methanesulfonate (EMS, Sigma‐Aldrich, Cat#M0880) for 4 h (M1 seeds). M1 plants were self‐pollinated to generate the M2 seeds. M2 seeds were harvested in pools of five plants. A total of 220 pools (1100 M2 families) and 216 plants per pool were screened for suppressors with reduced lobe phenotypes.

Mutants with repeatable phenotypes in M2 and M3 generations were backcrossed (BC) to *RCOg‐V*. BC1 F2 populations were used for mapping‐by‐sequencing based on the published guide (James et al., 2013). In each BC1F2 population (~220 plants), approximately 50 homozygous mutants showing reduced leaf lobe formation were selected, harvested (one leaf per sample), and pooled. Meanwhile, approximately 25 sister plants from the same BC1F2 population showing the *RCOg‐V* phenotype (deep lobes) were also harvested as a control pool. Pooled genomic DNA (gDNA) was extracted with a Qiagen DNeasy Plant Maxi Kit (Qiagen, Cat#68163), and further deep‐sequenced with an Illumina HiSeq3000 machine (paired‐end 150 bp reads, ~50× coverage for sample pools and ~25× coverage for control pools). Raw sequencing data were analyzed using the software SHOREmap v3.0 (Sun & Schneeberger, 2015) to identify all polymorphisms. Allele frequency was plotted by polymorphisms present in more than 50% of reads from the mutant pool (Figure 3B). Polymorphisms present in more than 50% of reads from the control pool were considered background mutations and excluded from further analysis, while polymorphisms present in >90% of reads from the mutant pool were used to determine mapping intervals and to identify candidates for the causal mutation. *slb59‐2* displayed a pin‐like inflorescence that is typical of *pin1* loss‐of‐function mutant; its mutation in *PIN1* was directly identified by Sanger sequencing.

### Allelism analysis

For allelism analysis, T‐DNA mutants of candidate genes were ordered from the Nottingham Arabidopsis Stock Centre (NASC): i.e., *pin1‐7* (*SALK_047613*) (Bilsborough et al., 2011), *cuc2‐3* (*SAIL_605_C09*) (Hibara et al., 2006), *cyp71‐2* (*SALK_050092*) (Li et al., 2007), *pgy1‐4*/*rpl10ab‐4* (*SALK_087642C*) (Horiguchi et al., 2011), *rpl34‐1* (*SALK_025693C*), and *nop2a‐2*/*oli2‐2* (*SALK_129648C*) (Fujikura et al., 2009). These T‐DNA mutants were then genotyped (primers and methods for genotyping are in Table S1) and crossed to the corresponding *slb* mutants from our suppressor screen. F1 plants were phenotyped for leaf shapes.

### Leaf shape analysis

Plant leaves were flattened onto white paper using transparent adhesive films, then digitally scanned at 800dpi resolution to obtain the silhouettes. To visualize size‐invariant symmetric variations in leaf blade shapes, leaf shape space principal component analysis was performed using the software Leaf Interrogator as described previously (Zhang et al., 2020); leaf dissection indexes were calculated with the formula [(perimeter)^2/(4*Pi*Area)]. The R package ggplot2 (Wickham, 2016) was applied to draw the box/point plots.

### Confocal microscopy

A Leica TCS‐SP8 upright confocal laser‐scanning microscope was used for confocal imaging. For Figure 1, imaging was performed using hybrid detectors (HyD) and a 25× water objective lens (N.A 0.95). Pixel format was set to 1024 × 1024. Sections were spaced 0.7–1 μm apart depending on the age of the leaf primordium. Scan speed was set to 400 Hz. Line averaging was set to 4 or 2 for imaging GFP and 1 or 2 for VENUS. GFP and VENUS were imaged using an argon laser, in a sequential scan, switching in between frames. GFP was excited using 488 nm and emission was collected within 493–510 nm. VENUS was excited using 514 nm and emission was collected within 520–550 nm. To mitigate cross‐talk between GFP and VENUS, channel dye separation was performed using the LAS AF software post image acquisition. Microscopy data were processed and visualized using the Imaris viewer (Oxford Instruments). For Figure 4, images were captured by an HC PL APO CS2 20×/0.75 IMM objective lens. Excitation was performed using an argon laser with 514 nm for VENUS and propidium iodide (PI). Images were collected at 520–550 nm for VENUS, 600–640 nm for PI, and 660–750 nm for chlorophyll auto‐fluorescence. Images were processed using Leica Application Suite X to generate maximum projections from representative confocal micrographs. For PI staining, dissected leaf primordia samples were stained in 0.1% PI (Sigma‐Aldrich, Cat#P4710) solution (in water) for 5 min, washed in water, then mounted on slides for imaging. To measure RCO‐VENUS fluorescence intensity (Figure 4K), leaf primordia of different genotypes were imaged with the same confocal microscope settings, then the fluorescence intensity in nuclei was measured using Fiji (Schindelin et al., 2012). For each sample, about 10 intact nuclei located in the center of the *RCO* expression domain were selected, which were round and similar in size to avoid possible confounding effects of cell cycle progression. The mean fluorescence intensity was then calculated. Results were plotted using the R package ggplot2 (Wickham, 2016).

### CRISPR/Cas9‐mediated mutagenesis in *Cardamine hirsuta*

CRISPR/Cas9‐mediated mutagenesis was used to generate *cyp71* mutants in *C. hirsuta* as described previously (Alvim Kamei et al., 2020). Two guide RNA (gRNA) sequences targeting *ChCYP71* were designed by CCTop (Stemmer et al., 2015) (https://cctop.cos.uni‐heidelberg.de/). Then two tandem *AtU6*_*pro*_ *:gRNA* cassettes were synthesized (Genscript Biotech) and inserted into a *pZP‐basta‐pDE‐Cas9* plasmid through LR reaction. The final *pZP‐basta‐pDE‐Cas9*‐*AtU6*_*pro*_ *:gRNA_ChCYP71* construct was transformed into *C. hirsuta* wild type (Oxford accession) using Agrobacteria GV3103 and by the floral dip method (Clough & Bent, 1998). Mutations in the targeted region were detected by PCR amplification and Sanger sequencing. T1 plants were screened with 0.2% Basta solution (Bayer). Basta‐resistant T1 plants with higher than 10% genome‐editing efficiency assessed by TIDE (Brinkman et al., 2014) (http://shinyapps.datacurators.nl/tide/) were selected to harvest T2 seeds. Homozygous mutants obtained in T2 or T3 generations were further genotyped to exclude those with the *Cas9* gene in the genome. Primers used for genotyping are listed in Table S1.

### Quantitative real‐time PCR

For the qPCR experiment shown in Figure 5G, *C. hirsuta* wild type (Oxford accession,Hay and Tsiantis 2006) and *chcyp71‐1* were cultivated on soil under long‐day conditions. Leaf primordia less than 800 μm long and the attached apical meristems from 17‐day‐old plants were dissected for RNA extraction. Three biological replicates per genotype (20 plant samples each replicate) were used. Total RNA was extracted using a Qiagen RNeasy Plant Mini Kit (Qiagen, Cat#74904) with on‐column DNase digestion (Qiagen Cat#79254), then reverse‐transcribed using a SuperScript VILO cDNA Synthesis Kit (Invitrogen, Cat#11754050) to generate the first‐strand cDNA. Quantitative PCR was performed in the QuantStudio 3 Real‐Time PCR System (ThermoFisher) using Power SYBR Green PCR Master Mix (ThermoFisher, Cat#4367659). *AtUBQ10* was used to normalize and quantify the expression of target genes. The primers used are listed in Table S1. Relative expression was calculated by 2^ΔΔCT^. qPCR results were plotted using the R package ggplot2 (Wickham, 2016).

### Quantification and statistical analysis

The numbers of samples and replicates that were analyzed are indicated in the figure legends. Statistical analyses were performed using R (R Core Team, 2022). For Figures 2C,D, and 4K, one‐way ANOVA combined with Tukey HSD tests was applied to test the differences among genotypes. For Figure 5G, a Welch two‐sample *t*‐test was applied. The significance threshold used was *P* < 0.05.

## Author Contributions

YW and MT designed experiments. YW performed most experiments and data analysis. NB performed experiments. YW, NB, and MT wrote the paper. MT designed and directed the study.

## Conflict of Interest

The authors declare no competing interests.

## Supporting information


**Table S1.** Oligonucleotides used in this study.
**Table S2.** Resources table.
**Figure S1.** Allelism test results of the *slb* mutants. Related to Table 1.
**Figure S2.** An inflorescence of *pin1‐12;RCOg‐V* (*slb59‐2*).

## Data Availability

The data that support the findings of this study are available on request from the corresponding author. The data are not publicly available due to privacy or ethical restrictions.
