# Supplementary material for: A suppressor screen of an Arabidopsis thaliana REDUCED COMPLEXITY (RCO)‐expressing strain provides insight into the genetics of leaf margin complexity
Source: Plant J. 2025 Jun 13;122(5):e70278. doi: 10.1111/tpj.70278 (PMC12165315; doi:10.1111/tpj.70278)
Supplement: Supplementary file 1 — Table S1. Oligonucleotides used in this study. Table S2. Resources table. Figure S1. Allelism test results of the slb mutants. Related to Table 1. Figure S2. An inflorescence of pin1‐12;RCOg‐V (slb59‐2). [file TPJ-122-0-s001.pdf]

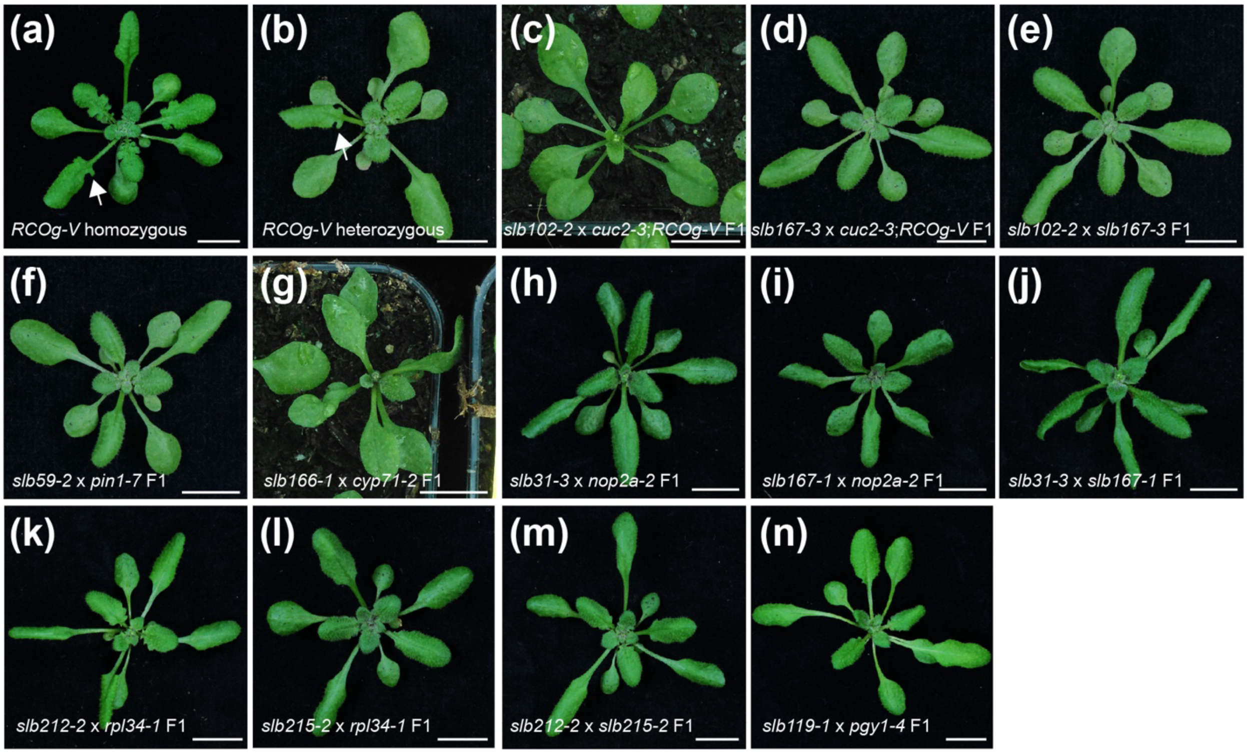

Figure S1. Allelism test results of the *slb* mutants. Related to Table 1.

Each *slb* mutant bears an EMS mutation and is homozygous for the *RCOg-V* transgene. In this allelism test, *slb* mutants were crossed with corresponding T-DNA insertion mutants lacking the *RCOg-V* transgene, except for *slb167-3* and *slb102-2*, which were crossed with the *cuc2-3;RCOg-V* double mutant. Some *slb* mutants were also crossed with each other to test whether the two were mutant alleles of the same gene. Rosettes of 3-week-old plants are presented (n=5~57 plants phenotyped for each genotype. Specific numbers are in Table 1).

(a) homozygous *RCOg-V*. White arrow indicates a deep lobe in a rosette leaf.

(b) A representative F1 plant from crosses of *RCOg-V* and Col-0. This heterozygous *RCOg-V* plant also produced deep lobes in rosette leaves (white arrow), therefore it could be compared to F1 plants in (f, g, h, i, k, l, and n).

(c) A representative F1 plant from crosses of *slb102-2* and *cuc2-3;RCOg-V*.

(d) A representative F1 plant from crosses of *slb167-3* and *cuc2-3;RCOg-V*.

(e) A representative F1 plant from crosses of *slb102-2* and *slb167-3*.

(f) A representative F1 plant from crosses of *slb59-2* and heterozygous *pin1-7*. The homozygous *pin1-7* mutant is infertile. *slb59-2* is fertile. This F1 plant was genotyped to confirm the T-DNA insertion and the *RCOg-V* transgene.

(g) A representative F1 plant from crosses of *slb166-1* and heterozygous *cyp71-2*. The homozygous *cyp71-2* mutant shows abnormal flowers and low fertility. *slb166-1* is fertile. This F1 plant was genotyped to confirm the T-DNA insertion and the *RCOg-V* transgene.

(h) A representative F1 plant from crosses of *slb31-3* and *nop2a-2*.

(i) A representative F1 plant from crosses of *slb167-1* and *nop2a-2*.

(j) A representative F1 plant from crosses of *slb31-3* and *slb167-1*.

(k) A representative F1 plant from crosses of *slb212-2* and *rpl34-1*.

(l) A representative F1 plant from crosses of *slb215-2* and *rpl34-1*.

(m) A representative F1 plant from crosses of *slb212-2* and *slb215-2*.

(n) A representative F1 plant from crosses of *slb119-1* and *pgy1-4*. Scale bars: 1 cm.

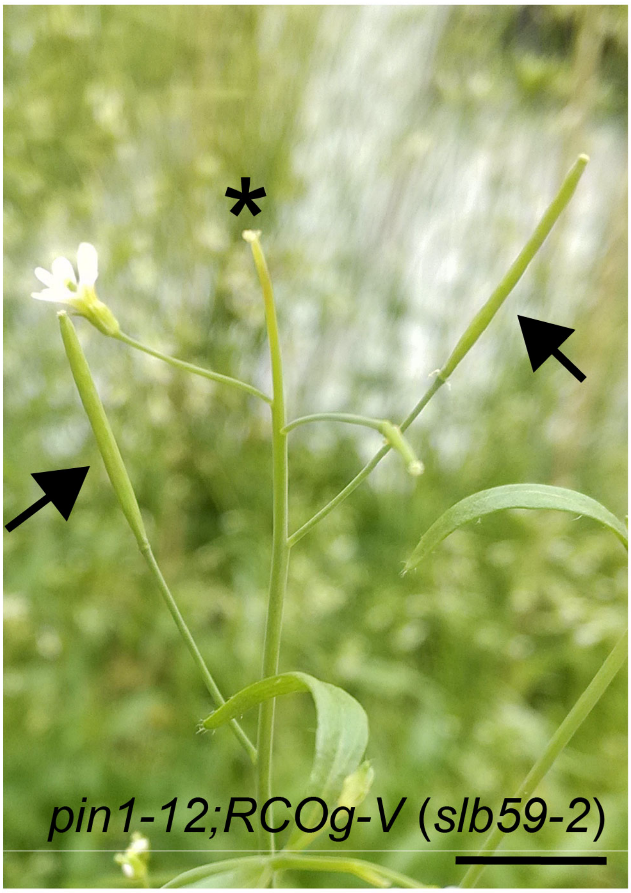

Figure S2. An inflorescence of *pin1-12;RCOg-V* (*slb59-2*).

pin1-12;RCOg-V (*slb59-2*) produces siliques with seeds (black arrows) and pin-like inflorescence (asterisk). Scale bar: 1 cm.

Table S1. Oligonucleotides used in this study.

Oligonucleotide name	Sequence (5'-3')	Usage
cyp71-2_gt_LP	CATTGGTGTTGTCGTGTTGTC	To genotype† *cyp71-2* (*SALK_050092*)
cyp71-2_gt_RP	CCTTCCAGAGAACGTGGTATG	
pin1-7_gt_LP	CAAAAACACCCCCAAAATTTC	To genotype *pin1-7* (*SALK_047613*)
pin1-7_gt_RP	AATCATCACAGCCACTGATCC	
cuc2-3_gt_LP	CAAGATTTGAAACAGCCTTATCG	To genotype *cuc2-3* (*SAIL_605_C09*)
cuc2-3_gt_RP	ATCCCAACAAGTGCACAAGTC	
pgy1-4_gt_LP	TGAAACTGTGTTGTCGTGGAG	To genotype *pgy1-4*/*rpl10ab-4* (*SALK_087642C*)
pgy1-4_gt_RP	AACATGGATGTTGAGGCTTTG	
rpl34-1_gt_LP	ACAACATCTGGAATCACCTCG	To genotype *rpl34-1* (*SALK_025693C*)
rpl34-1_gt_RP	GGCTGAAACAACTCCATCAAC	
nop2a-2_gt_LP	CATTTGTCCAGGAGCTTGAAG	To genotype *nop2a-2*/*oli2-2* (*SALK_129648C*)
nop2a-2_gt_RP	TTGTTTTACCACCAGGAGCAG	
JMLB1	GGCAATCAGCTGTTGCCCGTCTCACTGGTG	For SALK lines (*cyp71-2*, *pin1-7*, *pgy1-4*, *rpl34-1*, and *nop2a-2*)
SAIL_LB1	GCCTTTTCAGAAATGGATAAATAGCCTTGCTTCC	For SAIL lines (*cuc2-3*)
ChCYP71_gRNA_seq_F	CATCGAGATGTAGTTACACATG	To amplify sgRNA targeted region in *ChCYP71* for Sanger sequencing
ChCYP71_gRNA_seq_R	ATGTGTATCTATACAAACAAAG	
ChRCO_qPCR_F	GGTCAGGGTTTAATGAAGACGCAAACAAATC	For quantitative PCR to test RCO transcript levels
ChRCO_qPCR_R	GGAAAAGCCTGAGATATCGCCG	
ChUBQ10_qPCR_F	TGGTACTTTTGTGTGTTTTGAGGC	
ChUBQ10_qPCR_R	AAAGAGAGATAAGGACGCAAACATAGT	

†: We used LP (left primer) + RP (right primer) primers to amplify wild-type bands, RP + JMLB1 (SALK lines) or RP + SAIL_LB1 (SAIL lines) primers to amplify mutant bands. Specific primer pairs are as follows:

cyp71-2: cyp71-2_gt_LP + cyp71-2_gt_RP for the wild-type band; cyp71-2_gt_RP + JMLB1 for the mutant band.

pin1-7: pin1-7_gt_LP + pin1-7_gt_RP for the wild-type band; pin1-7_gt_RP + JMLB1 for the mutant band.

cuc2-3: cuc2-3_gt_LP + cuc2-3_gt_RP for the wild-type band; cuc2-3_gt_RP + SAIL_LB1 for the mutant band.

pgy1-4: pgy1-4_gt_LP + pgy1-4_gt_RP for the wild-type band; pgy1-4_gt_RP + JMLB1 for the mutant band.

rpl34-1: rpl34-1_gt_LP + rpl34-1_gt_RP for the wild-type band; rpl34-1_gt_RP + JMLB1 for the mutant band.

nop2a-2: nop2a-2_gt_LP + nop2a-2_gt_RP for the wild-type band; nop2a-2_gt_RP + JMLB1 for the mutant band.

Table S2 - RESOURCES TABLE

REAGENT or RESOURCE	SOURCE	IDENTIFIER
Chemicals, peptides, and recombinant proteins		
Ethyl methanesulfonate (EMS)	Sigma-Aldrich	Cat#M0880
BASTA	Bayer Crop Science	Cat#06470033
Mango Taq	Bioline	Cat#BIO-21083
Sucrose	Sigma-Aldrich	Cat#84097
MES	Roth	Cat#4256.4
Bacto Agar	Roth	Cat#5210.5
MS vitamins	Sigma-Aldrich	Cat#M3900
Murashige and Skoog basal salt mixture	Sigma-Aldrich	Cat#M5524
Propidium iodide (PI)	Sigma	Cat#P4710
Gateway LR Clonase II Enzyme mix	Invitrogen	Cat#1179102
Critical commercial assays		
RNeasy Plant Mini Kit	QIAGEN	Cat#74904
SuperScript VILO cDNA Synthesis Kit	Invitrogen	Cat#11754050
Power SYBR® Green PCR Master Mix	Thermo Fisher Scientific	Cat#4367659
DNeasy Plant Maxi Kit	QIAGEN	Cat#68163
Experimental models: Organisms/strains		
Plant genotypes	Sourse	More information
A. thaliana: wild type Col-0	NASC	N60000
A. thaliana: *ChRCO$_{pro}$::ChRCOg-VENUS* (*RCOg-V*)	this study	N/A

A. thaliana: *pAtPIN1::PIN1-GFP* (*PIN1-G*)	(Benková *et al.*, 2003)	N/A
A. thaliana: *RCOg-V*;*PIN1-G*	This study	by crossing *RCOg-V* and *PIN1-G*
A. thaliana: *pin1-12* (*slb59-2*)	This study	N/A
A. thaliana: *cuc2-4* (*slb102-2*)	This study	N/A
A. thaliana: *cuc2-5* (*slb167-3*)	This study	N/A
A. thaliana: *cyp71-3* (*slb166-1*)	This study	N/A
A. thaliana: *nop2a-5* (*slb31-3*)	This study	N/A
A. thaliana: *nop2a-6* (*slb167-1*)	This study	N/A
A. thaliana: *rpl34-2* (*slb212-2* and *slb215-2*)	This study	N/A
A. thaliana: *pgy1-5* (*slb119-1*)	This study	N/A
A. thaliana: *pin1-7*	(Bilsborough *et al.*, 2011)	*SALK_047613*
A. thaliana: *cuc2-3*	(Hibara *et al.*, 2006)	*SAIL_605_C09*
A. thaliana: *cuc2-3;RCOg-V*	This study	by crossing *cuc2-3* and *RCOg-V*
A. thaliana: *cyp71-2*	(Li *et al.*, 2007)	*SALK_050092*
A. thaliana: *pgy1-4*/*rpl10ab-4*	(Horiguchi *et al.*, 2011)	*SALK_087642C*
A. thaliana: *rpl34-1*	NASC	*SALK_025693C*
A. thaliana: *nop2a-2*/*oli2-2*	(Fujikura *et al.*, 2009)	*SALK_129648C*
C. hirsuta: wild type Oxford	N/A	N/A
C. hirsuta: *chcyp71-1*	This study	N/A
C. hirsuta: *chcyp71-2*	This study	N/A

Oligonucleotides		
All the oligonucleotides	This study	Table S1
Recombinant DNA		
pZP-basta-pDE-Cas9-AtU6$_{pro}$:gRNA_ChCYP71	This study	N/A
Software and algorithms		
SHOREmap (v3.0)	(Sun and Schneeberger, 2015)	https://bioinfo.mpipz.mpg.de/shoremap/
Leica application suite X	Leica	https://www.leica-microsystems.com/products/microscope-software/p/leica-las-x-ls/
Fiji (ImageJ 1.54f)	(Schindelin *et al.*, 2012)	https://fiji.sc/
Leaf Interrogator (LeafI)	(Zhang *et al.*, 2020)	https://gitlab.mpcdf.mpg.de/g-adamrunions/leafinterrogator_zhang_et_al
R (version 4.2.0)	(R Core Team, 2022)	https://www.r-project.org/
R package: ggplot2 (version 3.4.2)	(Wickham, 2016)	https://ggplot2.tidyverse.org/
CCTop	(Stemmer *et al.*, 2015)	https://cctop.cos.uni-heidelberg.de/
TIDE	(Brinkman *et al.*, 2014)	http://shinyapps.datacurators.nl/tide/
Imaris viewer	Oxford Instruments	https://imaris.oxinst.com/imaris-viewer
